# Amyand’s hernia, a rare finding for left incarcerated inguinal hernia in an infant: a case report

**DOI:** 10.1097/RC9.0000000000000357

**Published:** 2026-03-09

**Authors:** Florent Tshibwid A Zeng, Vincent de Paul Kaoma Cabala, Ildéphonse Teta Wa Mwanza, Pitchou Mukaz Mbey, Catherine Saleh Ugumba, Sébastien Mbuyi-Musanzayi

**Affiliations:** a Department of Surgery, Faculty of Medicine, Université de Lubumbashi, Lubumbashi, Democratic Republic of the Congo; b Department of Surgery, University Clinics of Lubumbashi, Lubumbashi, Democratic Republic of the Congo; c Department of Anesthesia and Resuscitation, Faculty of Medicine, Université de Lubumbashi, Lubumbashi, Democratic Republic of the Congo; d Department of Anesthesia and Resuscitation, University Clinics of Lubumbashi, Lubumbashi, Democratic Republic of the Congo

**Keywords:** Amyand’s hernia, appendectomy, case report, child, left-sided

## Abstract

**Introduction and clinical importance:**

Amyand’s hernia is a rare finding for pediatric inguinal hernia surgery. However, the left-sided location of Amyand’s hernia is extremely rare.

**Case presentation:**

We report the case of a 2-year-old patient who was admitted for an incarcerated left inguinoscrotal hernia with intraoperative diagnosis of Amyand’s hernia. Appendectomy and patent vaginalis ligation were done. Follow-up abdominal ultrasound and chest X-ray did not find midgut malrotation or situs inversus. Three months postoperatively, the patient is asymptomatic.

**Clinical discussion:**

In children presenting with a left incarcerated inguinal hernia, Amyand’s hernia remains a differential diagnosis. Thinking of it preoperatively will allow the surgeon to prepare for different intraoperative possibilities and act according to current recommendations in the pediatric population.

**Conclusion:**

Despite its rarity, left-sided Amyand’s hernia should be considered in an incarcerated left inguinal hernia.

## Introduction

In 1735, Claudius Amyand, a French surgeon, reported a surprising operative finding in an 11-year-old boy: a perforated vermiform appendix within the sac of a right inguinal hernia. He is credited with the first description of Amyand’s hernia (AH)[1]. Nowadays, this condition is defined by the presence of an appendix within an inguinal hernia sac, no matter whether the appendix is healthy or pathological[2]. Left-sided Amyand’s hernia (LSAH) represents less than 10% of all AH. This location may be linked to anomalies of rotation of the midgut or secondary to a mobile cecum, which is found in up to 20% of infants[3].

HIGHLIGHTS

- What is known on the subject: Amyand’s hernia is predominant on the right side due to the anatomical disposition of abdominal viscera. On rare occasions, left-sided Amyand’s hernia can occur.
- What this study adds: Left-sided Amyand’s hernia can present as an incarcerated hernia. It can also occur in patients with a right-sided appendix due to a mobile cecum.

Diagnosis of AH is mainly intraoperative, during a surgery for an irreducible inguinoscrotal hernia[2]. Its management is still controversial, especially when the appendix is healthy. However, Losanoff and Basson suggested surgical indications for AH, with a recent modification adapted to children by Jalil *et al*[2].

Following the SCARE guidelines[4], we report the case of a 24-month-old boy managed at our department (Table 1).

**Table 1:** Patient’s timeline.

Date	Step	Comments
May 13, 2025	Admission	Attendance of our department for a two-day painful groin swelling without vomiting.
May 15, 2025	Diagnosis	Clinical diagnosis of incarcerated left inguinal hernia and initiation of perioperative blood investigations.
May 15, 2025	Surgery	Emergency surgical procedure done three hours after admission.
May 20, 2025	Discharge	Patient discharged on postoperative day 5.
June 13, 2025	Follow-up	Investigation for malrotation with abdominal ultrasound.
August 14, 2025	Follow-up	Third month routine follow-up per institutional recommendations.

## Case presentation

A 24-month-old boy was brought by their parents for painful inguinoscrotal swelling evolving for 2 days. Parents initially gave oral paracetamol at home, without any improvement. The following day, swelling persisted, with cries, feeding refusal, but no vomiting. Stools were still passed. This led parents to consult our department for management. The patient was born from a full-term pregnancy, and weighed 3.100 g at birth. He had no other medical history.

On physical examination, the patient was lethargic, without fever (37.4°C), he had tachycardia (135 bpm) and polypnea (37 cpm), and moderate dehydration. He weighed 11 kg, which was normal for his sex and age. We identified a left inguinoscrotal painful swelling, tense, not expansive, neither impulsive, and irreducible. Abdominal examination was unremarkable, and cardiac sounds were normal. Diagnosis of left-sided incarcerated inguinal hernia was retained. The patient was prepared for an emergency open left herniotomy. A 8-French 8 Foley catheter was placed, and intravenous hydration with dextrose normal saline (DNS) at 6 ml/kg/hour and paracetamol (15 mg/kg/6 hours) was started. Blood electrolytes were within normal ranges, as well as an unremarkable full blood count.

**Figure 1.**
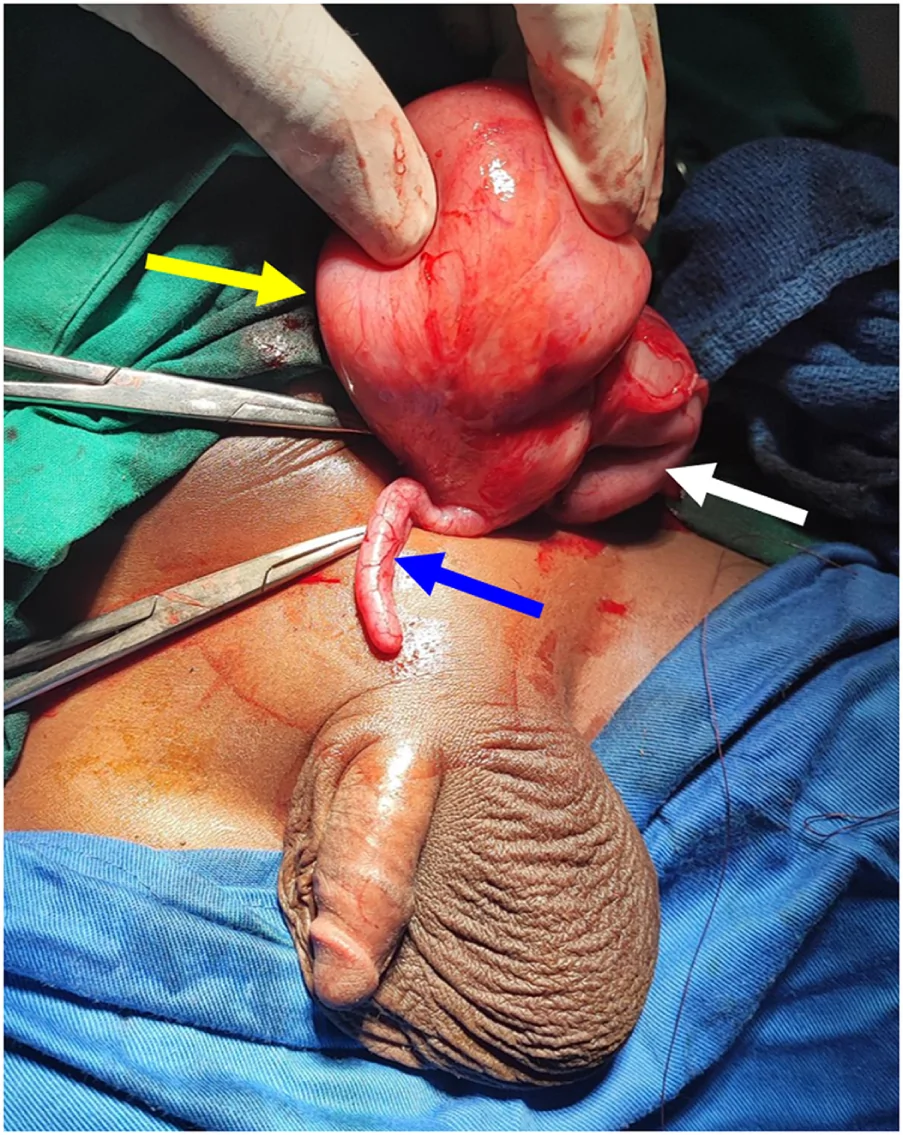
Intraoperative findings. Through inguinal incision, the opened patent vaginalis allowed to find the terminal ileum (white arrow), the cecum (yellow arrow) and the appendix (blue arrow) within it.

Three hours after admission, an emergency procedure was done by a general surgeon and a pediatric surgeon, both with 1 year of experience. In the operating room and under general anesthesia performed as follows: the induction was made with sevoflurane Mac 6% and propofol (4 mg/kg), followed by orotracheal intubation with a N°4 tube. Anesthesia maintenance was done with sevoflurane Mac 2 to 3%. Prophylaxis was done with Cefotaxime 100 mg/kg. During surgery, the patient was monitored with oxygen saturation, respiratory rate, heart rate, blood pressure, and temperature. All varied within normal ranges. A left 3-cm transverse inguinal incision was made without opening the aponeurosis, which allowed exposition of the hernia sac after deepening dissection beneath the fascia superficialis. The anterior aspect of the sac was opened, leading to identification of a healthy appendix, cecum, and terminal ileum within the hernia sac (Fig. 1). Due to the left position of the AH, we opted to proceed to an appendectomy, as recommended by a pediatric adaptation of the Losanoff-Basson classification[2]. We reduced the cecum without the need to extend the internal ring. We isolated elements of the spermatic cord and highly ligated the patent vaginalis using Polyglactin 3/0. The incision was closed in layers. Intravenous paracetamol was given at 15 mg/kg, and the sevoflurane was reduced from Mac 2.5% to nil, with subsequent patient waking. Aspiration of secretion in the intubation tube and the mouth was aspired with different probes, and extubation was done after oxygen arrest was well tolerated.

**Figure 2.**
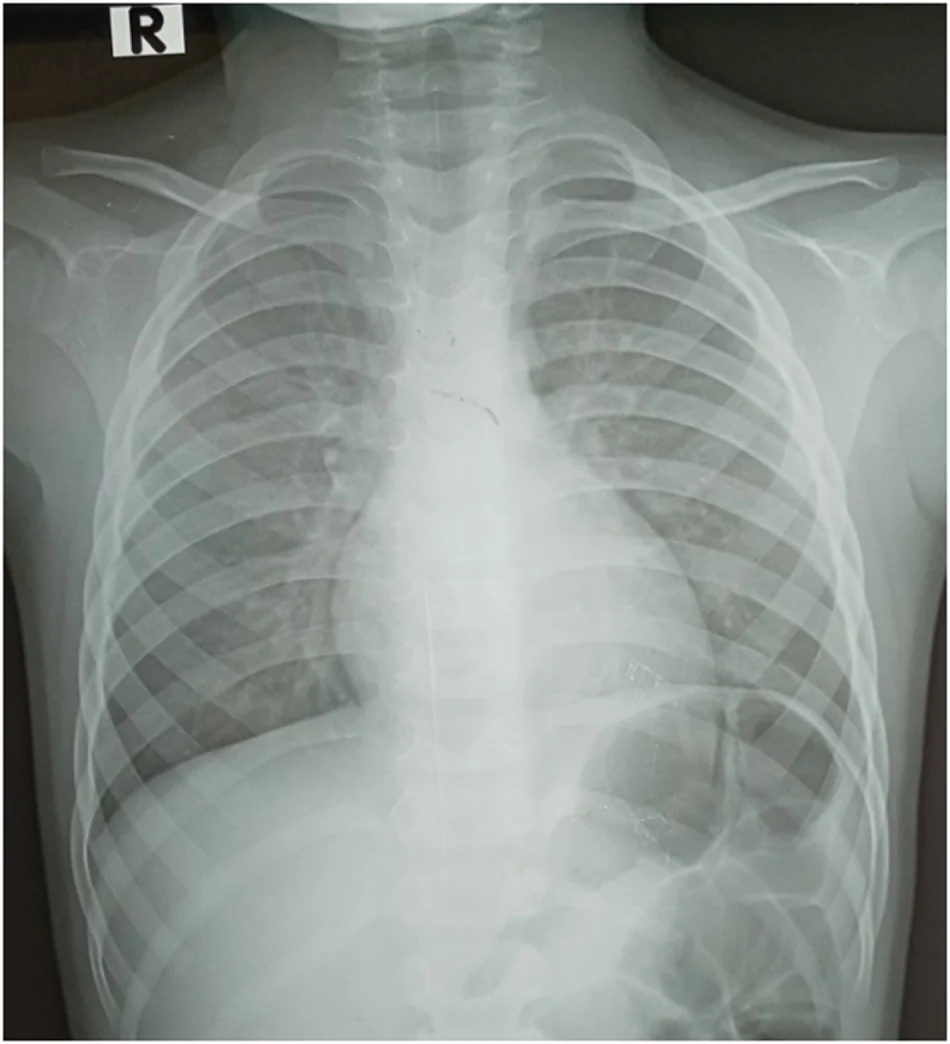
Chest X-ray. Note normal cardiac orientation excluding thoracic situs inversus.

The patient was kept in-hospital, with intravenous paracetamol (15 mg/kg/6 hours) and fluid maintenance with DNS in the immediate postoperative period at 4 ml/kg/hour. Oral feedings were started 12 hours postoperatively, without any complications. Per institutional guidelines, on postoperative day 5, he was discharged. Microscopic examination of the appendix showed normal histology, without inflammation or lymphoid hyperplasia. Postoperative ultrasound showed no sign of midgut malrotation (the superior mesenteric artery was left to the mesenteric vein), or situs inversus (the liver was right-sided), and the cecum was found lying in the right iliac fossa. A chest X-ray showed a normal-sided cardiac shadow (Fig. 2). Due to financial constraints, upper gastrointestinal series and contrast enema were not performed. Three months after surgery, clinical examination was unremarkable, and no sign of hernia recurrence was identified.

## Discussion

In this study, we reported a single case of incarcerated left inguinal hernia with an intraoperative finding of LSAH. With appendectomy and patent vaginalis high ligation, the postoperative course was free of any complication.

In the pediatric population, AH is a rarity, occurring in 0.01 to 0.42% of pediatric inguinal hernia^[^5–8^]^. It is mostly encountered in infants, with a median age around 1.5 years[5], but cases in older patients (6 and 4 years old) were reported^[^9,10^]^. Of all AH, left-sided ones occur in 6 to 10% of cases^[^5,6^]^. In the last 10 years, 18 cases of LSAH were reported (Table 2) ^[^9–26^]^, exclusivity occurring in males, mainly due to the predominance of inguinal hernia in boys, such as in our patient. Left-sided AH seems to occur in younger patients compared to all AH in general, with a median age at presentation of 9 months in our review. We believe that the occurrence of LSAH predominantly in infants is because a mobile caecum is documented in 20% of infants[3].

**Table 2:** Summary of recently reported pediatric left-sided Amyand’s hernia.

Authors	Age	Sex	Operative management	Healthy appendix	Associated anomalies	Outcome (follow-up duration)
Pilehroud RG *et al*, 2025	4 mo	M	Herniotomy with appendectomy	No	None (chest X-ray and abdominal US)	No complication (1 w)
Fopa PM *et al*, 2025	4 mo	M	Herniotomy with appendectomy	Yes	Not searched for	No complication (6 mo)
Mimouni M *et al*, 2024	3 mo	M	Herniotomy without appendectomy	Yes	Not searched for	No complication (5 mo)
Fikri E *et al*, 2024	1 y	M	Herniotomy without appendectomy	Yes	Not searched for	No complication (3 d)
Madany MEDM, 2024	18 mo	M	Herniotomy without appendectomy	Yes	None (Laparoscopy)	No complication (7 wks)
Sheng TS *et al*, 2023	1 y 4 mo	M	Herniotomy without appendectomy	Yes	None (chest X-ray)	No complication (6 mo)
Joshi *et al*, 2022	1 y 3 mo	M	Herniotomy without appendectomy	Yes	None (Upper GI series)	No complication (6 mo)
Kumar P *et al*, 2022	7 mo	M	Herniotomy without appendectomy	Yes	Not searched for	No complication (6 mo)
Ramu AH *et al*, 2021	2 y	M	Herniotomy without appendectomy	Yes	Not searched for	No complication (2.5 years)
Supangat *et al*, 2021	2 mo	M	Herniotomy with appendectomy	No	Not searched for	No complication (NR)
Kurahachi Y *et al*, 2021	10 mo	M	Herniotomy without appendectomy	Yes	None (undescribed)	No complication (NR)
Gopal M *et al*, 2021	11 d	M	Herniotomy with appendectomy	No	Not searched for	No complication (6 mo)
Byabene GD *et al*, 2020	8 mo	M	Herniotomy with appendectomy	Yes	Not searched for	No complication (3 mo)
Ogwuche EI *et al*, 2019	7 mo	M	Herniotomy with appendectomy	Yes	Not searched for	No complication (3 mo)
Velasquez-Bueso AE *et al*, 2019	6 yrs	M	Herniotomy with appendix inversion	Yes	Not searched for	No complication (NR)
Tripathi SP *et al*, 2018	18 mo	M	Herniotomy with appendectomy	Yes	None (chest x-ray and contrast enema)	No complication (6 w)
Bekele K *et al*, 2017	4 yrs	M	Herniotomy with appendectomy	No	Not searched for	No complication (6 mo)
Yoneyama F *et al*, 2015	8 mo	M	Herniotomy without appendectomy	Yes	None (chest X-ray and contrast enema)	No complication (1 y)

d: days, M: Male, mo: months, NR: Not reported, w(ks): week(s), y(rs): year(s)

Amyand’s hernia clinically presents as a usual pediatric hernia in most cases, with non-painful groin swelling. In a fourth of cases, AH presents as a painful, irreducible groin swelling, which was the case of our patient. Left-sided AH is thought to be secondary to anomalies of midgut rotation or fixation: mobile cecum, malrotation, or situs inversus[2]. In mobile cecum, the latter lies in the right iliac fossa, but Toldt’s fascia is poorly developed, allowing a variable degree of cecum mobility. In the highest grade, the cecum reaches the left abdomen, which can therefore lead to LSAH[27]. In malrotation, the cecum can be anywhere in the abdomen except in the right iliac fossa, depending on the exact type of malrotation. This may result in a lack of cecal attachments in some cases and lead to LSAH. In situs inversus, the cecum is in the left iliac fossa, making the appendix lie next to the left internal inguinal ring[3]. In our patient, no anatomical anomaly suggestive of malrotation or situs inversus was found postoperatively. We assumed the cause to be a mobile cecum. Among reviewed cases, predisposing anomalies were not searched for in 61% of cases. In the remaining cases, the means used to research these anomalies were a combination of chest X-ray, abdominal ultrasound, upper GI series, contrast enema, and surgical exploration, in a patient in whom laparoscopy was the surgical approach[15]. No rotational anomaly nor situs inversus was found in all six patients. Mobile cecum was assumed to be the cause^[^9,13–15,19,24,26^]^. In our patient, we retrospectively used chest X-ray to exclude situs inversus and abdominal ultrasound to exclude situs inversus and anomalies of rotation (malrotation, non-rotation, and reverse rotation). However, all rotational anomalies could not be excluded by abdominal ultrasound alone, and upper GI series and contrast enema were not done due to financial constraints.

With a clinical diagnosis of incarcerated inguinal hernia, imaging is not required in our practice. Emergency surgical repair is indicated to release the bowel as soon as possible, before vascular compromise. In institutions where ultrasound is readily available, it may be useful for preoperative diagnosis of AH, but it has less specificity. Additionally, computed tomography, which can provide a more precise preoperative diagnosis, is considered excessive for diagnosing AH[7].

Management of pediatric AH is made of herniotomy plus management of the appendix through the same incision. While the first is accepted by all, management of the normal-looking appendix is subject to controversy. Based on a literature review on pediatric Amyand’s hernia, Jalil *et al* proposed a pediatric adaptation of the Losanoff-Basson classification (Table 3)[2]. Among recent adaptations of the Losanoff-Basson classifications to children^[^2,28^]^, the one by Jalil *et al* is the only one taking into account the left-sided AH. But why is this important in clinical pediatric surgery? Rotational or fixation anomalies leading to a left-sided appendix are rare in children, and the first diagnosis hypothesis of painful left lower quadrant is not a left-sided appendicitis. Therefore, LSAH needed to be specifically addressed. Like other authors, we support appendectomy in LSAH with a healthy appendix due to the challenging diagnosis of potential acute appendicitis in case situs inversus or rotational anomalies are postoperatively confirmed^[^2,11^]^. Among the patients reviewed, 14 had a healthy appendix, and in five of them (35.7%), appendectomy was done^[^10,22–25^]^. This highlights persistent controversies in the management of the healthy appendix in left-sided AH. Authors supporting conservative management of the appendix advance that appendectomy: (a) may lead to abdominal contamination, (b) may weaken the abdominal wall due to additional dissection to accede to the appendiceal basis, and (c) the appendix may be useful later for Mitrofanoff or Malone procedure[11]. However, in our case, the cecum was part of the hernia content, thus no additional dissection was necessary to proceed with appendectomy. Moreover, follow-up of patients in whom appendectomy was not performed in LSAH is quite short (3 days to 12 months)^[^11–17,19,20,27^]^, not allowing formal exclusion of appendicitis occurrence when such patients get older, and then evaluation of delayed diagnosis due to atypical presentation. In our opinion, larger and multicentric studies with longer patient follow-up should be conducted to determine the best option for healthy appendix management in pediatric LSAH.

**Table 3:** **Pediatric adaptation of Losanoff-Basson classification and management of Amyand’s hernia by Jalil et al**[2].

Classification	Management
Right inguinal hernia sac containing normal appendix	Appendix reduction and open/laparoscopic hernia repair
Right inguinal hernia with acute appendix without abdominal sepsis and/or dense adhesion of appendix with hernial sac Left inguinal hernia with or without acute appendicitis	Appendectomy and hernia repair through open or laparoscopic approach
Inguinal hernia and complicated acute appendicitis within the hernial sac	Appendectomy through laparotomy or laparoscopy and hernia repair
Amyand’s Hernia with acute appendicitis associated with concomitant abdominal pathology	Laparotomy or laparoscopy with appendectomy, with management of concomitant disease
Recurrent inguinal hernia with adhesion of appendix with hernia sac	Appendectomy, herniotomy with narrowing of deep ring and post wall repair if needed

Postoperative course is reported to be uneventf ul in most cases, with no recurrence of inguinal hernia and no development of acute appendicitis in patients without appendectomy^[^6,8^]^. In our case, no complication was noted either.

## Strengths and limitations

We reported a patient with a rare diagnosis who was managed in line with current recommendations. The main limitation is the lack of preoperative diagnosis.

## Patient perspective

The patient’s parents were satisfied with the outcome of surgical treatment, without any complications.

## Conclusion

In infants, owing to a mobile cecum, left-sided Amyand’s hernia can be exceptionally encountered. It can present as an incarcerated inguinal hernia, whose management is a surgical emergency. Management of a healthy appendix in such cases is still controversial, but should be guided by larger studies evaluating the benefits and risks of performing appendectomy in such cases. In our case, we endorsed the recent adaptation of the Losanoff-Basson classification for the pediatric population by Jalil *et al*, with good outcomes.
